# Complete Genome and Methylome Sequences of *Salmonella enterica* subsp. *enterica* Serovars Typhimurium, Saintpaul, and Stanleyville from the SARA/SARB Collection

**DOI:** 10.1128/genomeA.00031-17

**Published:** 2017-03-16

**Authors:** Kuan Yao, Richard J. Roberts, Marc W. Allard, Maria Hoffmann

**Affiliations:** a Division of Microbiology, Office of Regulatory Science, Center for Food Safety and Nutrition, U.S. Food and Drug Administration, College Park, Maryland, USA; b School of Systems Biology, George Mason University, Manassas, Virginia, USA; c New England Biolabs, Inc., Ipswich, Massachusetts, USA

## Abstract

In this announcement, we report the complete genome and methylome sequences of three *Salmonella enterica* strains from the SARA and SARB collection: *S. enterica* subsp. *enterica* serovar Typhimurium (SARA13), *S. enterica* subsp. *enterica* serovar Saintpaul (SARA26), and *S. enterica* subsp. *enterica* serovar Stanleyville (SARB61).

## GENOME ANNOUNCEMENT

The three *Salmonella* strains included in this study—*S. enterica* subsp. *enterica* serovars Typhimurium, Saintpaul, and Stanleyville—belong to the Salmonella Reference Collection SARA/SARB (1, 2). The reference collection was established based on the genetic structure of *S. enterica* characterized by multilocus enzyme electrophoresis (MEE) (3). *S*. Typhimurium is the most common cause of food poisoning in the United States, and outbreaks have been linked to poultry, beef products, and rodents. The prevalence of this serotype has increased from 9% to 33% since 1990 (4). *S*. Saintpaul is closely related to *S*. Typhimurium (5), with infections having resulted from consumption of several fresh produce commodities such as cucumbers, jalapeño peppers, and tomatoes (6). *S.* Stanleyville (SARB61) belongs to the SARB collection, which encompasses a more genetically diverse group of *S. enterica* that are commonly found in the environment, animals, and humans (3). Multiple cases of *S*. Stanleyville were reported in Cameroon, Mali, and Uganda (7, 8). Between 2003 and 2013, there were 65 cases of multistate *S*. Stanleyville infections reported in the United States (9).

*S*. Typhimurium, Saintpaul, and Stanleyville isolates were sequenced based on previously published procedures (10, 11). The continuous long-read data were *de novo* assembled using the PacBio Hierarchical Genome Assembly Process (HGAP) version 3.0 (12). The assembled sequences were annotated using the NCBI Prokaryotic Genome Annotation Pipeline (PGAP) and deposited at DDBJ/EMBL/GenBank.

The complete *S*. Saintpaul (SARA26) genome was sequenced with 125× coverage. The complete genome size was 4,686,793 bp with a G/C content of 52.02%. *S*. Saintpaul consisted of 4,491 genes. The PHAge Search Tool (PHAST) analysis for prophage sequence detection did not identify any intact phages (13). The *S*. Typhimurium genome was sequenced with 171× coverage. The closed genome for the chromosome was 4,819,807 bp and 93,826 bp for the plasmid. The genome consisted of 4,770 genes with a G+C content of 52.21% for the chromosome and 53.1% for the plasmid. Using PHAST analysis, prophages Gifsy-1, Gifsy-2, and Salmon-ST64B were identified. The *S*. Stanleyville genome was sequenced with 144× coverage. The genome consisted of 4,888,463 bp for the chromosome and three plasmids with sizes of 106,449 bp, 58,302 bp, and 49,762 bp. The complete genome contained 4,991 genes. The G+C content for the chromosome was 52.13%; the G+C content ranging from the largest to the smallest plasmid was 51.0%, 52.1%, and 52.0%, respectively. PHAGE analysis indicated the presence of prophage Salmon-SPN1S.

The DNA methyltransferase activities were analyzed based on the kinetic variations of the nucleotide incorporation rate of the PacBio RSII sequencing platform (14). The single-molecule real-time data of the methylomes are summarized in Table 1. They are also deposited in REBASE (15) as follows: *S*. Saintpaul, http://rebase.neb.com/cgi-bin/pacbioget?20626; *S*. Typhimurium, http://rebase.neb.com/cgi-bin/pacbioget?20625; and *S*. Stanleyville, http://rebase.neb.com/cgi-bin/pacbioget?20799. While most of the motifs have been found in other *Salmonella* strains, the motif ACRCAG found as the recognition sequence of a type IIG restriction/modification enzyme is new and unique.

**TABLE 1:** Summary of active methylases and their recognition sequences

Strain	Assignment	Methyltransferase specificity	Methylation type	Restriction modification type
*S*. Saintpaul	M.SenSARA26Dam	GATC	m6A	Orphan alpha
	M.SenSARA26I	CAGAG	m6A	III beta
	M.SenSARA26II	ATGCAT	m6A	II beta
	SenSARA26III	ACRCAG	m6A	II G,S, alpha
*S*. Typhimurium	M.Sen13Dcm	CCWGG	m5C	Orphan
	M.Sen13I	GAGNNNNNNRTAYG	m6A	I gamma
	M.Sen13II	CAGAG	m6A	III beta
	M.Sen13IV	ATGCAT	m6A	II beta
	Sen13III	GATCAG	m6A	II G,S, alpha
*S*. Stanleyville	M.Sen624I	CAGAG	m6A	III beta
	M.Sen624II	GAGNNNNNNRTAYG	m6A	I gamma
	M.Sen624III	ATGCAT	m6A	II beta

### Accession number(s).

Sequences have been deposited in GenBank under the following accession numbers: *S*. Saintpaul, CP017727; *S*. Typhimurium, CP017728 and CP017729; and *S*. Stanleyville, CP017723, CP017724, CP017725, and CP017726.
